# 2-Chloro-*N*-(3-methyl­phen­yl)benzamide

**DOI:** 10.1107/S1600536812005739

**Published:** 2012-02-17

**Authors:** Vinola Z. Rodrigues, B. Thimme Gowda, Viktor Vrábel, Jozef Kožíšek

**Affiliations:** a Department of Chemistry, Mangalore University, Mangalagangotri-574 199, Mangalore, India; b Institute of Physical Chemistry and Chemical Physics, Slovak University of Technology, Radlinského 9, SK-812 37 Bratislava, Slovak Republic

## Abstract

In the structure of the title compound, C_14_H_12_ClNO, the *ortho*-Cl atom in the benzoyl ring is positioned *syn* to the C=O bond, while the *meta*-methyl group in the aniline ring is positioned *anti* to the N—H bond. The amide group forms dihedral angles of 60.1 (1) and 22.0 (1)°, respectively, with the benzoyl and aniline rings, while the angle between these rings is 38.7 (1)°. The crystal structure is stabilized by N—H⋯O hydrogen bonds, which give rise to infinite chains running along the *c* axis.

## Related literature

For studies, including ours, on the effects of substituents on the structures and other aspects of *N*-(ar­yl)-amides, see: Bowes *et al.* (2003 ▶); Gowda *et al.* (1999 ▶, 2006 ▶); Rodrigues *et al.* (2011 ▶); Saeed *et al.* (2010 ▶); for *N*-(ar­yl)-methane­sulfonamides, see: Gowda *et al.* (2007 ▶); for *N*-chloro­aryl­amides, see: Jyothi & Gowda (2004 ▶); and for *N*-bromo­aryl­sulfonamides, see: Usha & Gowda (2006 ▶).
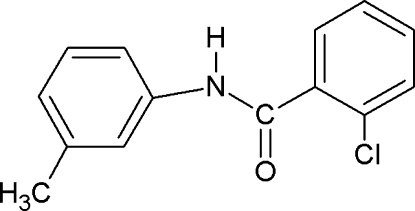


## Experimental

### 

#### Crystal data

C_14_H_12_ClNO
*M* *_r_* = 245.70
Tetragonal, 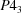

*a* = 8.8751 (3) Å
*c* = 15.9642 (5) Å
*V* = 1257.45 (6) Å^3^
*Z* = 4
Mo *K*α radiation
μ = 0.29 mm^−1^
*T* = 295 K
0.4 × 0.3 × 0.2 mm

#### Data collection

Oxford Diffraction Xcalibur System diffractometer
Absorption correction: multi-scan (*CrysAlis RED*; Oxford Diffraction, 2009 ▶) *T*_min_ = 0.898, *T*_max_ = 0.942
8244 measured reflections
2552 independent reflections
1757 reflections with *I* > 2σ(*I*)
*R*_int_ = 0.019

#### Refinement

*R*[*F* ^2^ > 2σ(*F* ^2^)] = 0.031
*wR*(*F* ^2^) = 0.069
*S* = 1.04
2552 reflections
158 parameters
2 restraints
H atoms treated by a mixture of independent and constrained refinement
Δρ_max_ = 0.08 e Å^−3^
Δρ_min_ = −0.11 e Å^−3^
Absolute structure: Flack (1983 ▶), 1229 Friedel pairs
Flack parameter: 0.00 (6)

### 

Data collection: *CrysAlis CCD* (Oxford Diffraction, 2009 ▶); cell refinement: *CrysAlis CCD*; data reduction: *CrysAlis RED* (Oxford Diffraction, 2009 ▶); program(s) used to solve structure: *SHELXS97* (Sheldrick, 2008 ▶); program(s) used to refine structure: *SHELXL97* (Sheldrick, 2008 ▶); molecular graphics: *DIAMOND* (Brandenburg, 2002 ▶); software used to prepare material for publication: *SHELXL97*, *PLATON* (Spek, 2009 ▶) and *WinGX* (Farrugia, 1999 ▶).

## Supplementary Material

Crystal structure: contains datablock(s) I, global. DOI: 10.1107/S1600536812005739/sj5196sup1.cif

Structure factors: contains datablock(s) I. DOI: 10.1107/S1600536812005739/sj5196Isup2.hkl

Supplementary material file. DOI: 10.1107/S1600536812005739/sj5196Isup3.cml

Additional supplementary materials:  crystallographic information; 3D view; checkCIF report

## Figures and Tables

**Table 1:** Hydrogen-bond geometry (Å, °)

*D*—H⋯*A*	*D*—H	H⋯*A*	*D*⋯*A*	*D*—H⋯*A*
N1—H1⋯O1^i^	0.86 (1)	2.03 (1)	2.8790 (16)	171 (2)

Symmetry code: (i) 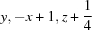.

**Fig. 1.**
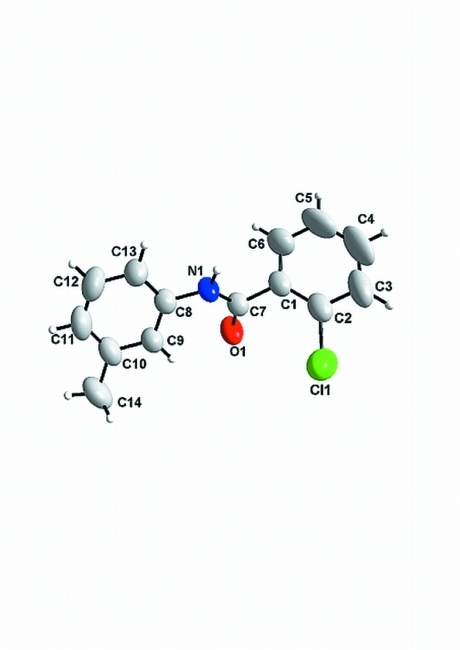
Molecular structure of the title compound showing the atom labelling scheme. Displacement ellipsoids are drawn at the 50% probability level. H atoms are represented as small spheres of arbitrary radius.

**Fig. 2.**
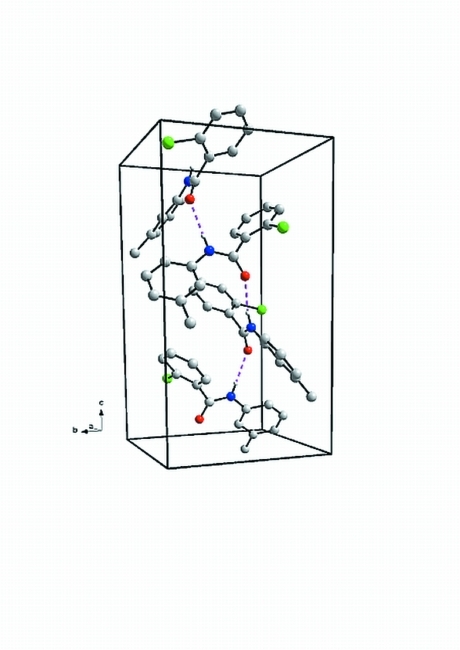
Packing view of the title compound. Molecular chains along along the c axis are generated by N—H···O hydrogen bonds which are shown as dashed lines. H atoms have been omitted.

## supplementary crystallographic information

### Comment

The amide and sulfonamide moieties are the constituents of many biologically important compounds. As part of our studies on the substituent effects on the structures and other aspects of *N*-(aryl)-amides (Bowes *et al.*, 2003; Gowda *et al.*, 1999, 2006; Rodrigues *et al.*, 2011; Saeed *et al.*, 2010), *N*-(aryl)-methanesulfonamides (Gowda *et al.*, 2007), *N*-chloroarylsulfonamides (Jyothi & Gowda, 2004) and *N*-bromoarylsulfonamides (Usha & Gowda, 2006), in the present work, the crystal structure of 2-chloro-*N*-(3-methylphenyl)benzamide (I) has been determined (Fig.1).

In (I), the *ortho*-Cl atom in the benzoyl ring is positioned *syn* to the C=O bond, while the *meta*-methyl group in the anilino ring is positioned *anti* to the N—H bond, similar to that observed in 3-chloro-*N*-(3-methylphenyl)benzamide (I) (Rodrigues *et al.*, 2011).

The amide group forms dihedral angles of 60.1 (1) and 22.0 (1)°, respectively, with the benzoyl and aniline rings, while the angle between the benzoyl and aniline rings is 38.7 (1)°, compared to the value of 77.4 (1)° in (II).

In the crystal structure, intermolecular N1—H1···O1 hydrogen bonds (Table 1) link the molecules into infinite chains running along the *c*-axis. Part of the crystal structure is shown in Fig. 2.

### Experimental

The title compound was prepared by a method similar to the one described by Gowda *et al.* (2006). The purity of the compound was checked by determining its melting point. It was characterized by recording its infrared and NMR spectra.

Plate like colorless single crystals of the title compound used in the X-ray diffraction studies were obtained by slow evaporation of an ethanol solution of the compound (0.5 g in about 30 ml of ethanol) at room temperature.

### Refinement

All hydrogen atoms bound to carbon were placed in calculated positions with C–H distances of 0.93Å (C-aromatic), 0.96Å (*C*-methyl) and constrained to ride on their parent atoms. The amide H atom was located in a difference map and refined with the N—H distance restrained to 0.86 (1) Å. *U*_iso_(H) values were set at 1.2 *U*_eq_(C-aromatic, N) and 1.5 *U*_eq_(*C*-methyl).

### Crystal data

**Table:**

C_14_H_12_ClNO	*D*_x_ = 1.298 Mg m^−^^3^
*M**_r_* = 245.70	Mo *K*α radiation, λ = 0.71073 Å
Tetragonal, *P*4_3_	Cell parameters from 2552 reflections
*a* = 8.8751 (3) Å	θ = 3.4–29.3°
*c* = 15.9642 (5) Å	µ = 0.29 mm^−^^1^
*V* = 1257.45 (6) Å^3^	*T* = 295 K
*Z* = 4	Plate, colourless
*F*(000) = 512	0.4 × 0.3 × 0.2 mm

### Data collection

**Table:**

Oxford Diffraction Xcalibur System diffractometer	2552 independent reflections
Radiation source: fine-focus sealed tube	1757 reflections with *I* > 2σ(*I*)
Graphite monochromator	*R*_int_ = 0.019
Detector resolution: 0 pixels mm^-1^	θ_max_ = 26.4°, θ_min_ = 4.1°
ω scans with κ offsets	*h* = −10→11
Absorption correction: multi-scan (*CrysAlis RED*; Oxford Diffraction, 2009)	*k* = −11→10
*T*_min_ = 0.898, *T*_max_ = 0.942	*l* = −19→19
8244 measured reflections	

### Refinement

**Table:**

Refinement on *F*^2^	Secondary atom site location: difference Fourier map
Least-squares matrix: full	Hydrogen site location: inferred from neighbouring sites
*R*[*F*^2^ > 2σ(*F*^2^)] = 0.031	H atoms treated by a mixture of independent and constrained refinement
*wR*(*F*^2^) = 0.069	*w* = 1/[σ^2^(*F*_o_^2^) + (0.0348*P*)^2^] where *P* = (*F*_o_^2^ + 2*F*_c_^2^)/3
*S* = 1.04	(Δ/σ)_max_ < 0.001
2552 reflections	Δρ_max_ = 0.08 e Å^−^^3^
158 parameters	Δρ_min_ = −0.11 e Å^−^^3^
2 restraints	Absolute structure: Flack (1983), ???? Friedel pairs
Primary atom site location: structure-invariant direct methods	Flack parameter: 0.00 (6)

### Special details

**Table:**

Geometry. All e.s.d.'s (except the e.s.d. in the dihedral angle between two l.s. planes) are estimated using the full covariance matrix. The cell e.s.d.'s are taken into account individually in the estimation of e.s.d.'s in distances, angles and torsion angles; correlations between e.s.d.'s in cell parameters are only used when they are defined by crystal symmetry. An approximate (isotropic) treatment of cell e.s.d.'s is used for estimating e.s.d.'s involving l.s. planes.
Refinement. Refinement of *F*^2^ against ALL reflections. The weighted *R*-factor *wR* and goodness of fit *S* are based on *F*^2^, conventional *R*-factors *R* are based on *F*, with *F* set to zero for negative *F*^2^. The threshold expression of *F*^2^ > σ(*F*^2^) is used only for calculating *R*-factors(gt) *etc*. and is not relevant to the choice of reflections for refinement. *R*-factors based on *F*^2^ are statistically about twice as large as those based on *F*, and *R*- factors based on ALL data will be even larger.

### Fractional atomic coordinates and isotropic or equivalent isotropic displacement parameters (Å^2^)

**Table:**

	*x*	*y*	*z*	*U*_iso_*/*U*_eq_	
C1	0.6060 (2)	0.6255 (2)	0.21616 (9)	0.0579 (4)	
C2	0.6965 (2)	0.7340 (2)	0.25296 (11)	0.0731 (5)	
C3	0.6388 (4)	0.8363 (2)	0.30991 (13)	0.1015 (8)	
H3	0.7002	0.9103	0.3330	0.122*	
C4	0.4900 (5)	0.8268 (3)	0.33167 (17)	0.1176 (10)	
H4	0.4505	0.8952	0.3700	0.141*	
C5	0.3986 (3)	0.7188 (4)	0.29813 (18)	0.1124 (9)	
H5	0.2979	0.7131	0.3140	0.135*	
C6	0.4562 (3)	0.6180 (2)	0.24059 (14)	0.0819 (6)	
H6	0.3939	0.5442	0.2180	0.098*	
C7	0.66413 (19)	0.52431 (19)	0.14825 (9)	0.0525 (4)	
C8	0.69202 (18)	0.25396 (19)	0.11204 (9)	0.0529 (4)	
C9	0.7955 (2)	0.2664 (2)	0.04811 (10)	0.0582 (4)	
H9	0.8393	0.3594	0.0368	0.070*	
C10	0.8357 (2)	0.1411 (2)	−0.00002 (10)	0.0678 (5)	
C11	0.7689 (3)	0.0059 (3)	0.01807 (13)	0.0839 (6)	
H11	0.7941	−0.0786	−0.0134	0.101*	
C12	0.6663 (3)	−0.0075 (2)	0.08115 (15)	0.0927 (7)	
H12	0.6228	−0.1007	0.0923	0.111*	
C13	0.6264 (2)	0.1167 (2)	0.12884 (12)	0.0732 (5)	
H13	0.5560	0.1075	0.1717	0.088*	
C14	0.9504 (3)	0.1563 (3)	−0.06817 (13)	0.0986 (7)	
H14A	0.9645	0.0605	−0.0950	0.148*	
H14B	1.0442	0.1896	−0.0447	0.148*	
H14C	0.9160	0.2285	−0.1086	0.148*	
N1	0.65297 (16)	0.37678 (15)	0.16483 (7)	0.0541 (3)	
H1	0.6216 (16)	0.3554 (18)	0.2143 (4)	0.057 (5)*	
O1	0.71348 (15)	0.57708 (12)	0.08283 (6)	0.0695 (3)	
Cl1	0.88575 (8)	0.74156 (9)	0.22927 (4)	0.1238 (3)	

### Atomic displacement parameters (Å^2^)

**Table:**

	*U*^11^	*U*^22^	*U*^33^	*U*^12^	*U*^13^	*U*^23^
C1	0.0768 (13)	0.0532 (10)	0.0435 (9)	0.0070 (9)	0.0009 (8)	0.0051 (8)
C2	0.1011 (15)	0.0678 (12)	0.0505 (10)	−0.0026 (11)	0.0069 (10)	−0.0031 (9)
C3	0.167 (3)	0.0738 (15)	0.0636 (13)	−0.0023 (15)	0.0112 (15)	−0.0163 (12)
C4	0.184 (3)	0.0806 (18)	0.0882 (17)	0.052 (2)	0.024 (2)	−0.0110 (15)
C5	0.106 (2)	0.122 (2)	0.1091 (19)	0.0456 (19)	0.0298 (16)	−0.0028 (18)
C6	0.0827 (15)	0.0798 (14)	0.0831 (13)	0.0154 (11)	0.0071 (12)	−0.0038 (12)
C7	0.0604 (10)	0.0576 (11)	0.0397 (8)	0.0039 (8)	−0.0057 (7)	0.0000 (8)
C8	0.0618 (11)	0.0561 (11)	0.0407 (8)	0.0074 (9)	−0.0086 (8)	0.0017 (7)
C9	0.0647 (11)	0.0597 (11)	0.0503 (9)	0.0074 (8)	−0.0004 (8)	−0.0018 (8)
C10	0.0762 (13)	0.0770 (14)	0.0504 (10)	0.0228 (11)	−0.0066 (9)	−0.0098 (9)
C11	0.1158 (18)	0.0646 (14)	0.0714 (13)	0.0147 (12)	−0.0142 (13)	−0.0211 (10)
C12	0.134 (2)	0.0550 (13)	0.0897 (15)	−0.0091 (12)	−0.0063 (15)	−0.0062 (12)
C13	0.0945 (15)	0.0604 (13)	0.0647 (11)	−0.0058 (10)	0.0061 (10)	0.0001 (10)
C14	0.1021 (18)	0.118 (2)	0.0757 (13)	0.0372 (14)	0.0136 (12)	−0.0137 (13)
N1	0.0747 (10)	0.0516 (9)	0.0361 (7)	0.0009 (7)	0.0059 (6)	0.0023 (6)
O1	0.1070 (10)	0.0619 (8)	0.0397 (6)	−0.0015 (6)	0.0060 (6)	0.0053 (6)
Cl1	0.1097 (5)	0.1673 (7)	0.0943 (4)	−0.0532 (4)	0.0089 (3)	−0.0407 (4)

### Geometric parameters (Å, º)

**Table:**

C1—C2	1.384 (3)	C8—C9	1.378 (2)
C1—C6	1.387 (3)	C8—N1	1.421 (2)
C1—C7	1.499 (2)	C9—C10	1.398 (2)
C2—C3	1.383 (3)	C9—H9	0.9300
C2—Cl1	1.723 (2)	C10—C11	1.369 (3)
C3—C4	1.368 (4)	C10—C14	1.496 (3)
C3—H3	0.9300	C11—C12	1.363 (3)
C4—C5	1.366 (4)	C11—H11	0.9300
C4—H4	0.9300	C12—C13	1.386 (3)
C5—C6	1.381 (3)	C12—H12	0.9300
C5—H5	0.9300	C13—H13	0.9300
C6—H6	0.9300	C14—H14A	0.9600
C7—O1	1.2254 (19)	C14—H14B	0.9600
C7—N1	1.340 (2)	C14—H14C	0.9600
C8—C13	1.376 (2)	N1—H1	0.859 (2)
			
C2—C1—C6	118.06 (17)	C8—C9—C10	120.92 (17)
C2—C1—C7	121.60 (16)	C8—C9—H9	119.5
C6—C1—C7	120.25 (17)	C10—C9—H9	119.5
C3—C2—C1	121.4 (2)	C11—C10—C9	118.07 (18)
C3—C2—Cl1	118.66 (19)	C11—C10—C14	121.81 (18)
C1—C2—Cl1	119.96 (14)	C9—C10—C14	120.12 (19)
C4—C3—C2	119.0 (2)	C12—C11—C10	121.44 (18)
C4—C3—H3	120.5	C12—C11—H11	119.3
C2—C3—H3	120.5	C10—C11—H11	119.3
C5—C4—C3	121.1 (2)	C11—C12—C13	120.5 (2)
C5—C4—H4	119.4	C11—C12—H12	119.7
C3—C4—H4	119.4	C13—C12—H12	119.7
C4—C5—C6	119.7 (3)	C8—C13—C12	119.23 (18)
C4—C5—H5	120.2	C8—C13—H13	120.4
C6—C5—H5	120.2	C12—C13—H13	120.4
C5—C6—C1	120.7 (2)	C10—C14—H14A	109.5
C5—C6—H6	119.6	C10—C14—H14B	109.5
C1—C6—H6	119.6	H14A—C14—H14B	109.5
O1—C7—N1	124.64 (14)	C10—C14—H14C	109.5
O1—C7—C1	120.67 (15)	H14A—C14—H14C	109.5
N1—C7—C1	114.67 (14)	H14B—C14—H14C	109.5
C13—C8—C9	119.83 (16)	C7—N1—C8	127.93 (13)
C13—C8—N1	117.39 (15)	C7—N1—H1	115.0 (11)
C9—C8—N1	122.74 (16)	C8—N1—H1	117.1 (11)
			
C6—C1—C2—C3	−2.6 (3)	C13—C8—C9—C10	−0.3 (2)
C7—C1—C2—C3	173.88 (17)	N1—C8—C9—C10	177.13 (14)
C6—C1—C2—Cl1	176.38 (15)	C8—C9—C10—C11	0.2 (2)
C7—C1—C2—Cl1	−7.1 (2)	C8—C9—C10—C14	−178.91 (17)
C1—C2—C3—C4	1.8 (3)	C9—C10—C11—C12	−0.2 (3)
Cl1—C2—C3—C4	−177.27 (19)	C14—C10—C11—C12	178.9 (2)
C2—C3—C4—C5	−0.1 (4)	C10—C11—C12—C13	0.2 (3)
C3—C4—C5—C6	−0.7 (4)	C9—C8—C13—C12	0.3 (3)
C4—C5—C6—C1	−0.3 (4)	N1—C8—C13—C12	−177.23 (16)
C2—C1—C6—C5	1.9 (3)	C11—C12—C13—C8	−0.3 (3)
C7—C1—C6—C5	−174.69 (19)	O1—C7—N1—C8	−1.0 (3)
C2—C1—C7—O1	−58.9 (2)	C1—C7—N1—C8	177.16 (15)
C6—C1—C7—O1	117.55 (19)	C13—C8—N1—C7	−158.57 (16)
C2—C1—C7—N1	122.85 (17)	C9—C8—N1—C7	24.0 (2)
C6—C1—C7—N1	−60.7 (2)		

### Hydrogen-bond geometry (Å, º)

**Table:**

*D*—H···*A*	*D*—H	H···*A*	*D*···*A*	*D*—H···*A*
N1—H1···O1^i^	0.86 (1)	2.03 (1)	2.8790 (16)	171 (2)

Symmetry code: (i) *y*, −*x*+1, *z*+1/4.
